# Supplementary figures and images for: Potential hidden Plasmodium vivax malaria reservoirs from low parasitemia Duffy-negative Ethiopians: Molecular evidence
Source: PLoS Negl Trop Dis. 2023 Jul 3;17(7):e0011326. doi: 10.1371/journal.pntd.0011326 (PMC10348516; doi:10.1371/journal.pntd.0011326)

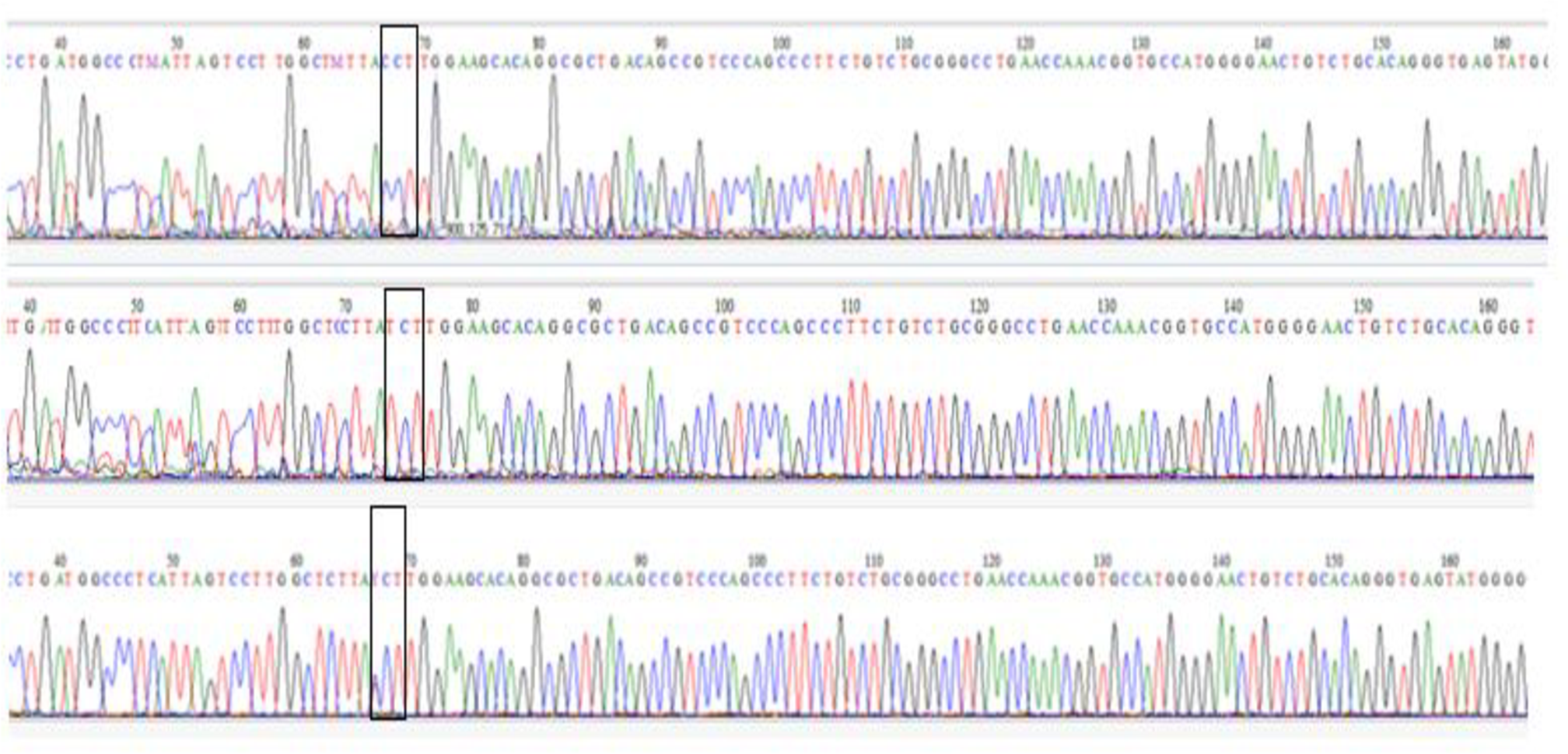

Supplement: S1 Appendix — (TIF) [file pntd.0011326.s001.tif]

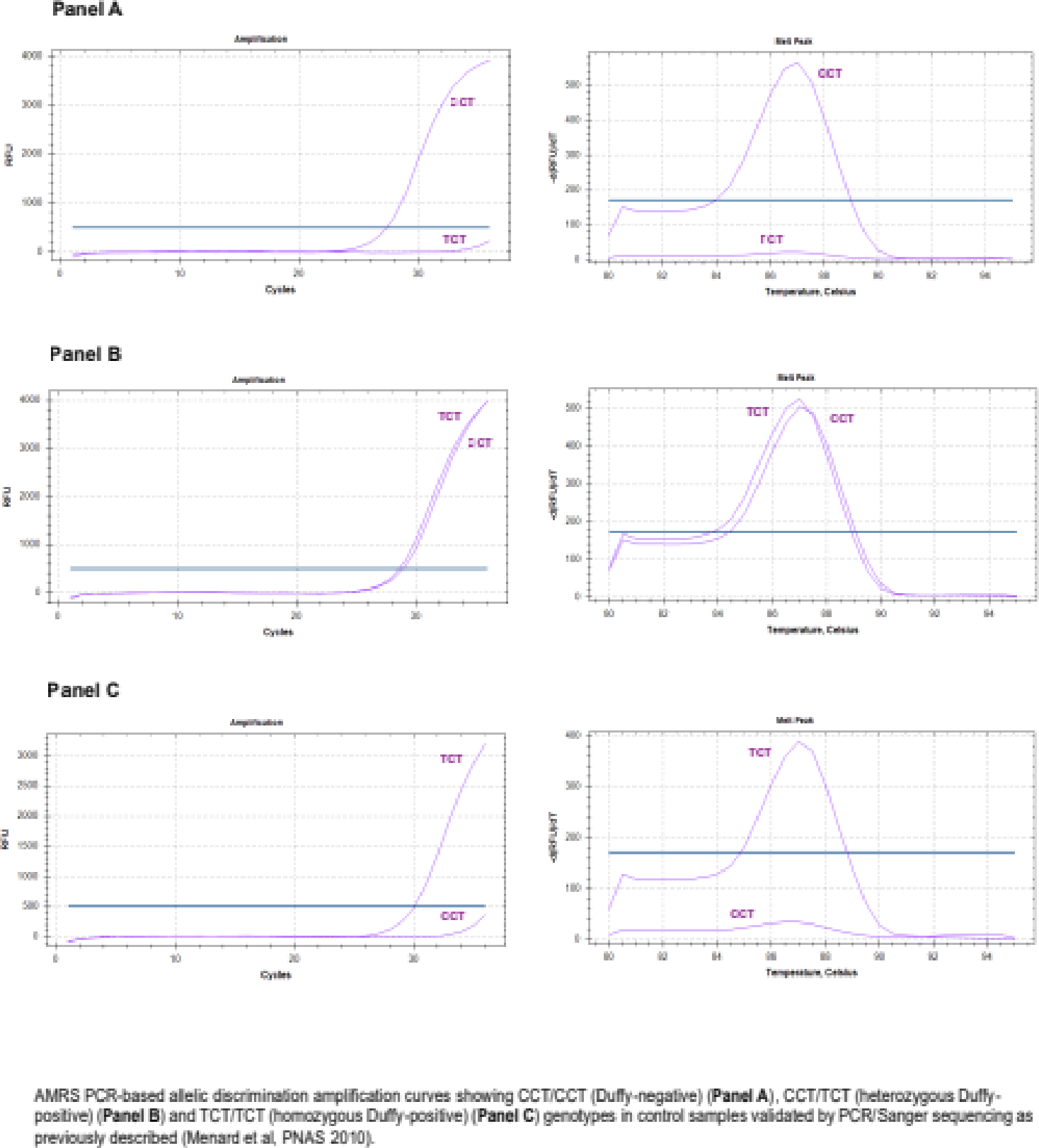

Supplement: S2 Appendix — (TIF) [file pntd.0011326.s002.tif]
