# Supplementary material for: Potential hidden Plasmodium vivax malaria reservoirs from low parasitemia Duffy-negative Ethiopians: Molecular evidence
Source: PLoS Negl Trop Dis. 2023 Jul 3;17(7):e0011326. doi: 10.1371/journal.pntd.0011326 (PMC10348516; doi:10.1371/journal.pntd.0011326)
Supplement: Attachment — Submitted filename: renamed_f7530.docx [file pntd.0011326.s003.docx]

**Author’s Response for Editors**

1) In the online submission form you indicate that your data is available from a co-author on this manuscript. According to our Data Policy, data must either be included with the submission, be placed in a public repository,or available from a third party contact point who must not be an author on the manuscript. Please revise your data statement to a non-author institutional point of contact, such as a data access or ethics committee, and send this to us via return email. Please also include contact information for the third party organization (this contact should be a central address and not a named person), and please include the full citation of where the data can be found.

**Author’s response:** We appreciate the editor for the remark, on the online submission we have indicated that “all relevant data are within the manuscript. The data that support the findings of this study are available from the **corresponding author**”. It is not from third party or co-authors.

2) We do not publish any copyright or trademark symbols that usually accompany proprietary names, eg (R), (C), or TM  (e.g. next to drug or reagent names). Please remove all instances of trademark/copyright symbols throughout the text, including Whatman™ on line no 173,CFX96™ on line no 191.

**Author’s response:** We thank the editors for the comments. All comments were corrected in the revised manuscript per the suggestion.

3) Please upload all main figures (Figure1 to Figure3) as separate Figure files in .tif or .eps format. For more information about how to convert and format your figure files please see our guidelines:[https://journals.plos.org/plosntds/s/figures](https://journals.plos.org/plosntds/s/figures)

**Author’s response:** We appreciate the editor for the remark. All the figures (Figure1 to Figure3) were uploaded as a separate figure files in .tif format.

4) We note that you have uploaded "Ethical Clearance" as supporting information file . Please change the file type to " Other" as Ethical Clearance should not be uploaded as supporting information files.

**Author’s response:** We thank the editor for the comments. Ethical Clearance re-uploaded as file type " Other" per the direction given.

5) Some material included in your submission may be copyrighted. According to PLOS’s copyright policy, authors who use figures or other material (e.g., graphics, clipart, maps) from another author or copyright holder must demonstrate or obtain permission to publish this material under the Creative Commons Attribution 4.0 International (CC BY 4.0) License used by PLOS journals. Please closely review the details of PLOS’s copyright requirements here: PLOS Licenses and Copyright. If you need to request permissions from a copyright holder, you may use PLOS's Copyright Content Permission form.

Please respond directly to this email and provide any known details concerning your material's license terms and permissions required for reuse, even if you have not yet obtained copyright permissions or are unsure of your material's copyright compatibility. Once you have responded and addressed all other outstanding technical requirements, you may resubmit your manuscript within Editorial Manager.

Potential Copyright Issues:

Figure [1,2]: please (a) provide a direct link to the base layer of the map (i.e., the country or region border shape) and ensure this is also included in the figure legend; and (b) provide a link to the terms of use / license information for the base layer image or shapefile. We cannot publish proprietary or copyrighted maps (e.g. Google Maps, Mapquest) and the terms of use for your map base layer must be compatible with our CC BY 4.0 license.

Note: if you created the map in a software program like R or ArcGIS, please locate and indicate the source of the basemap shapefile onto which data has been plotted.

**Author’s response:** We thank the editor for raising the point. The maps we have used were not obtained from a copyrighted source and not published elsewhere previously. All the maps used were created using ArcGIS software program, and the base layer of the maps were uploaded and submitted as file type " Other". The maps were plotted from eth_admbnda_adm3_csa_bofed_20201027 basemap shapefile.
